# Whole genome co-expression analysis of soybean cytochrome P450 genes identifies nodulation-specific P450 monooxygenases

**DOI:** 10.1186/1471-2229-10-243

**Published:** 2010-11-09

**Authors:** Satish K Guttikonda, Joshi Trupti, Naveen C Bisht, Hui Chen, Yong-Qiang C An, Sona Pandey, Dong Xu, Oliver Yu

**Affiliations:** 1 Donald Danforth Plant Science Center, St Louis, MO 63132, USA; 2 Digital Biology Laboratory, Computer Science Department and Christopher S. Bond Life Sciences Center, University of Missouri, Columbia, MO 65211, USA; 3 Plant Genetics Research Unit, ARS-USDA, Donald Danforth Plant Science Center, 975 N. Warson Road, St. Louis, MO 63132, USA

## Abstract

**Background:**

Cytochrome P450 monooxygenases (P450s) catalyze oxidation of various substrates using oxygen and NAD(P)H. Plant P450s are involved in the biosynthesis of primary and secondary metabolites performing diverse biological functions. The recent availability of the soybean genome sequence allows us to identify and analyze soybean putative P450s at a genome scale. Co-expression analysis using an available soybean microarray and Illumina sequencing data provides clues for functional annotation of these enzymes. This approach is based on the assumption that genes that have similar expression patterns across a set of conditions may have a functional relationship.

**Results:**

We have identified a total number of 332 full-length P450 genes and 378 pseudogenes from the soybean genome. From the full-length sequences, 195 genes belong to A-type, which could be further divided into 20 families. The remaining 137 genes belong to non-A type P450s and are classified into 28 families. A total of 178 probe sets were found to correspond to P450 genes on the Affymetrix soybean array. Out of these probe sets, 108 represented single genes. Using the 28 publicly available microarray libraries that contain organ-specific information, some tissue-specific P450s were identified. Similarly, stress responsive soybean P450s were retrieved from 99 microarray soybean libraries. We also utilized Illumina transcriptome sequencing technology to analyze the expressions of all 332 soybean P450 genes. This dataset contains total RNAs isolated from nodules, roots, root tips, leaves, flowers, green pods, apical meristem, mock-inoculated and *Bradyrhizobium japonicum*-infected root hair cells. The tissue-specific expression patterns of these P450 genes were analyzed and the expression of a representative set of genes were confirmed by qRT-PCR. We performed the co-expression analysis on many of the 108 P450 genes on the Affymetrix arrays. First we confirmed that *CYP93C5 *(an isoflavone synthase gene) is co-expressed with several genes encoding isoflavonoid-related metabolic enzymes. We then focused on nodulation-induced P450s and found that *CYP728H1 *was co-expressed with the genes involved in phenylpropanoid metabolism. Similarly, *CYP736A34 *was highly co-expressed with lipoxygenase, lectin and *CYP83D1*, all of which are involved in root and nodule development.

**Conclusions:**

The genome scale analysis of P450s in soybean reveals many unique features of these important enzymes in this crop although the functions of most of them are largely unknown. Gene co-expression analysis proves to be a useful tool to infer the function of uncharacterized genes. Our work presented here could provide important leads toward functional genomics studies of soybean P450s and their regulatory network through the integration of reverse genetics, biochemistry, and metabolic profiling tools. The identification of nodule-specific P450s and their further exploitation may help us to better understand the intriguing process of soybean and rhizobium interaction.

## Background

Cytochrome P450 monooxygenases (P450s) are enzymes found in most organisms from bacteria, to plants and human [1]. They catalyze the oxidation of various substrates using oxygen and NAD(P)H. In plants, large numbers of P450 genes form complex super-families and play important roles in many plant metabolic processes. They are involved in biosynthesis of pigments (anthocyanins), accessory pigments (carotenoids), defense-related compounds (some phytoalexins), UV protectants (flavonoids and sinapoyl esters), structural polymers (lignins), and fatty acids. P450s also contribute to the homeostasis of signalling molecules such as plant hormones because they are frequently the rate-limiting enzymes of hormone biosyntheses [2]. Similar to their functions in animals, P450s are also responsible for degradation of endogenous as well as exogenous compounds such as herbicides, insecticides and pollutants [3].

Structurally, the bacterial P450s are soluble proteins. In contrast, all plant P450s studied so far are membrane-localized. Most of them are anchored on the cytoplasmic surface of the endoplasmic reticulum (ER) by a hydrophobic peptide present at the N-terminus, possibly forming a trans-membrane segment [4]. Analysis of the Arabidopsis and other plant P450 sequences predicts potential signal peptides that should target some of the P450s to the plastids or to the mitochondria [2], though no mitochondrial P450s are known in plants. The number of P450 genes in plants is much higher than in other organisms, correlating to the fact that plants produce a huge repertoire of primary and secondary metabolites. For nomenclature and classification of diverse P450 genes, a universal system has been set up based on the protein sequence identity and phylogeny [5]. Briefly, P450 proteins that share at least 40% identity are assigned to the same family. They are further grouped into sub-families that share at least 55% identity. A few exceptions to this nomenclature system do occur, especially in plants where gene duplication events make it more complex. In such cases, phylogeny and gene organization are taken as criteria for family assignment. P450 genes for all organisms are named and classified by a P450 nomenclature committee in chronological order of sequence submission (David Nelson: dnelson@uthsc.edu). To distinguish from other organisms, plant P450s are classified into families from CYP71A1 to CYP99XY, and then from CYP701A1 and above [2]. P450s in plants are traditionally classified in two types: the A-type and the non-A type [6,7]. Recently, plant P450s have been re-classified into 11 clans. The A-type is now grouped as the CYP71 clan and the non-A type has 10 clans including the CYP51, CYP72, CYP74, CYP85, CYP86, CYP97, CYP710, CYP711, CYP727, and CYP746 clan [7].

Here, we carried out a systematic analysis of the soybean genome for P450 gene families. Soybean (*Glycine max*) is one of the most important legume species and a leading oilseed crop in the world. Processed soybeans are the largest source of vegetable oil and protein feed. According to a USDA report, soybean represented 56% of world oilseed production in 2008 http://www.soystats.com/2009/. The soybean genome has recently been sequenced and various large-scale expression analyses have been established in soybean, providing unique resources for genomic analysis of this important gene family [8]. Importantly, the soybean P450 information can be compared to other fully sequenced plant genomes. As of September 2010, from the fully sequenced plant genomes, there are 71 full-length P450 genes in moss *Physcomitrella patens*, 245 in *Arabidopsis*, 332 in rice, and 310 in poplar [9].

Since membrane-bound enzymes are traditionally more difficult to study, few P450s have been functionally characterized. In the *Arabidopsis *genome, only 41 of the 245 coding sequences have been associated with a specific biochemical function [10]. Recently, a large-scale P450 co-expression analysis with functional annotation of the 245 *Arabidopsis *P450 genes was performed to predict the function of unknown P450 genes [11]. This co-expression analysis revealed expression patterns of the majority of *Arabidopsis *P450s, and provided novel clues on individual P450 functions, pathways, and their regulatory networks. A novel phenolic pathway in pollen development was identified based on this co-expression analysis [12].

In this study, using *in silico *resources and bioinformatics tools, we identified and annotated putative functional P450 encoding sequences in the soybean genome. Phylogenetic analysis using amino acid sequences allowed us to identify gene orthologs and clusters of orthologous groups for further characterizations. By analyzing large-scale microarray and Illumina sequencing data, we also analyzed the co-expression of P450 genes in soybean, which could provide important clues to their function. We were able to identify tissue-specific P450 genes that may play roles in biological process like nodulation, floral development, and seed maturation. The large-scale expression analyses were confirmed for selected P450 genes using quantitative real-time reverse-transcription polymerase chain reaction (qRT-PCR).

## Results and Discussions

### Soybean cytochrome P450 genes

A large number of soybean P450 genes have been deposited into the cytochrome P450 database http://drnelson.uthsc.edu/CytochromeP450.html following the completion of the soybean genome sequencing. A total of 332 full-length soybean P450 genes and 378 pseudogenes with Glyma location markers were retrieved from the database. To screen for additional P450 genes, we performed BLAST search using standard P450 domains against Phytozome 4.0 and confirmed all 332 putative P450 genes in the genome. No new full-length P450 gene was discovered by three sets of domain search algorithms, including Pfam, Panther, and KOG. Additionally, we were able to identify 13 new pseudogenes from the domain searches, which were later confirmed and classified by the P450 nomenclature committee and updated in the cytochrome P450 database (as of September, 2010). Currently, the cytochrome P450 database lists all the soybean P450 genes, pseudogenes, and their corresponding Glyma location markers. These genes are classified into A-type and Non-A type P450s. There are 20 families of the A-type and 28 families of the non-A type P450s in soybean, consisting of 195 and 137 full-length sequences, respectively (Tables 1 and 2).

**Table 1:** Comparison of A-type P450 families among soybean, *Medicago*, *Arabidopsis*, rice, poplar, grape and moss.

Family	Soybean	Medicago	Arabidopsis	Rice	Popular	Grape	Moss
A-Type							
*CYP71 Clan*							
CYP71	55	37	52	84	25	24	0
CYP73	3	1	1	3	3	3	4
CYP75	7	0	1	3	3	11	0
CYP76	14	6	8	29	13	24	0
CYP77	4	2	5	2	3	2	0
CYP78	11	1	6	8	10	7	3
CYP79	5	3	7	4	4	9	0
CYP80	0	0	0	0	6	6	0
CYP81	12	5	18	12	28	21	0
CYP82	24	10	5	0	10	34	0
CYP83	12	9	1	0	5	0	0
CYP84	3	3	2	3	3	3	0
CYP89	8	9	7	14	10	14	0
**CYP92**	**2**	**1**	**0**	**9**	**8**	**6**	**0**
CYP93	13	8	1	3	4	4	0
CYP98	2	1	3	2	5	1	1
CYP99	0	0	0	2	0	0	0
CYP701	2	1	1	5	1	1	1
CYP703	1	1	1	1	1	1	3
**CYP705**	**0**	**0**	**26**	**0**	**0**	**0**	**0**
CYP706	3	1	7	4	5	9	0
CYP712	2	1	2	0	9	2	0
CYP723	0	0	0	2	0	0	0
**CYP736**	**12**	**1**	**0**	**0**	**6**	**8**	**0**
Others	0	0	0	0	0	0	29

**Total**	**195**	**101**	**154**	**190**	**162**	**166**	**41**

The families highlighted in bold have unique distributions among these species as described in the text.

**Table 2:** Comparison of non-A type P450 families among soybean, *Medicago*, *Arabidopsis*, rice, poplar, grape and moss.

Family	Soybean	Medicago	Arabidopsis	Rice	Popular	Grape	Moss
Non A-Type							
***CYP51 clan***							
CYP51	2	1	1	10	2	2	1
***CYP72 clan***							
CYP72	12	7	9	13	6	22	0
**CYP709**	**0**	**1**	**3**	**9**	**1**	**1**	**0**
CYP714	6	3	2	5	6	6	0
CYP715	6	1	1	1	2	1	0
CYP721	2	1	1	2	6	5	0
CYP734	3	1	1	4	2	2	0
CYP735	3	1	1	2	2	1	0
CYP749	0	0	0	0	9	0	0
***CYP74 clan***							
CYP74	6	4	2	4	6	7	3
***CYP85 clan***							
CYP85	5	1	2	1	3	2	0
CYP87	2	2	1	11	12	7	0
CYP88	3	3	2	1	2	2	0
CYP90	12	4	4	5	7	4	0
**CYP702**	**0**	**0**	**6**	**0**	**0**	**0**	**0**
CYP707	10	3	4	3	7	5	0
**CYP708**	**0**	**0**	**4**	**0**	**0**	**0**	**0**
CYP716	7	3	2	0	17	15	1
CYP718	1	0	1	0	17	15	1
CYP720	2	1	1	0	1	1	0
CYP722	2	1	1	1	1	1	0
CYP724	1	0	1	1	2	2	0
**CYP728**	**2**	**0**	**0**	**11**	**7**	**6**	**0**
CYP729	0	1	0	2	1	0	0
**CYP733**	**3**	**0**	**0**	**1**	**1**	**1**	**0**
***CYP86 clan***							
CYP86	9	3	11	5	8	6	2
CYP94	14	4	6	18	13	9	2
CYP96	7	5	13	12	9	5	0
CYP704	5	14	3	7	6	6	6
***CYP97 clan***							
CYP97	5	4	3	3	3	3	3
***CYP710 clan***							
CYP710	2	1	4	4	1	1	2
***CYP711 clan***							
CYP711	4	2	1	5	2	1	0
***CYP727 clan***							
**CYP727**	**1**	**0**	**0**	**1**	**2**	**0**	**0**
***CYP746 clan***							
CYP746	0	0	0	0	0	0	1
**Others**	0	0	0	0	0	0	9

**Total**	**137**	**72**	**91**	**142**	**148**	**124**	**30**

Non-A type P450 families are organized into 10 clans. The families highlighted in bold have unique distributions among these species as described in the text.

Among these families, CYP71 is the largest A-type family, with 55 members (Table 1); while CYP94 is the largest non-A type family (Table 2), with 14 members. Only four families; CYP703, 718, 724, and 727 consist of a single gene. The remaining 44 families are all multi-gene families most likely due to two genome duplication events in soybean [8,13]. The number of pseudogenes matched well with the number of full-length genes in the A-type families (Additional File 1, Table S1A). For example, CYP71, the largest family also has 65 pseudogenes, the most in all families. In contrast, pseudugene numbers did not match the numbers of full-length genes in non-A type families (Additional File 1, Table S1B). For example, CYP710 has only two full-length genes but 17 pseudogenes. We speculate that non-A type P450s are evolutionally more ancient than A-type families, allowing more time for gene duplication and rearrangement, resulting in more diverse compositions than A-type genes.

The soybean P450 genes were compared to a few selected plant species including *Medicago*, *Arabidopsis*, rice, poplar, grape and moss (Tables 1 and 2). A comparison of P450s among soybean, *Medicago*, *Arabidopsis *and rice, where comprehensive analyses of the P450 genes have been published, revealed that the CYP92 family is present in most of the higher plants except *Arabidopsis*. CYP92A6 is proposed to be involved in brassinosteroid (BR) biosynthesis during etiolated hypocotyl growth of pea [14,15]. Since this family is absent in *Arabidopsis *that has a robust BR biosynthesis and signaling system, CYP92A6 might have additional functions that need to be evaluated. Similarly, CYP727, CYP728 and CYP733 families are present in rice and soybean and absent in *Arabidopsis *and *Medicago*. For example, CYP727A1 is represented by a single gene in rice and CYP727B5 is a single gene in soybean. These are probably solo-function genes similar to the CYP51G family [9]. CYP736 family is present in soybean and *Medicago *[16] but absent from - *Arabidopsis *and rice. However, like several other families, the CYP736 family genes are also found in grape and poplar (Table 1). We did not identify any unique "legume-specific" A-type P450s. In general, moss has the most distinct P450 family distribution, representing a significant evolutionary distance.

The CYP702 and CYP708 of non-A type families are present in *Arabidopsis *but absent in soybean, *Medicago *or rice (Table 2). The P450 genes from these two families are all unique to *Arabidopsis *and its closest relatives, such *as Brassica napus*, making these the only known Brassicaceae-specific CYP proteins thus far. Among them, CYP708A2 is a thalianol hydroxylase, and CYP702A2 and CYP702A3 are triterpene synthases [17]. In contrast, if grape and poplar P450s are included, we cannot find any P450 families that are unique to soybean, or unique to legumes (soybean and *Medicago *combined). Even though legumes P450 are involved in making unique compounds, such as isoflavones (CYP93C), these P450 genes are not distant enough to form their own families.

### Phylogenetic analysis of soybean and *Arabidopsis *P450 genes

A neighbor-joining (N-J) phylogenetic tree for P450 protein sequences from soybean and *Arabidopsis *was constructed to determine the orthologous genes and cluster groups between these two species using P-distance in the MEGA4 package [18]. The soybean P450 genes were first classified into two major classes, A-type and non-A-type (Additional File 2, Figure S1A and Figure S1B). The A-type genes contain 59% of the soybean sequences (195 of the 332 sequences) and represent many of the plant-specific enzymes for the synthesis of secondary products (such as phenylpropanoids, etc.). The proteins encoded by the non-A-type sequences contain 137 of the 332 sequences and include enzymes involved in the synthesis of primary metabolic compounds (such as sterols, fatty acids, etc.) hormones and other signaling molecules. Based on the phylogenetic tree, CYP736 family that is present in soybean and *Medicago *closely resembles to the CYP83 and CYP81 gene families in *Arabidopsis *(Additional File 2, Figure S1A). The CYP83 family genes are involved in glucosinolate metabolism [19]. As shown in Additional File 2, Figure S1A, the CYP733A family in soybean and CYP81K in *Arabidopsis *belong to the same cluster, but no function has been assigned to either of these two gene families.

**Figure 1:**
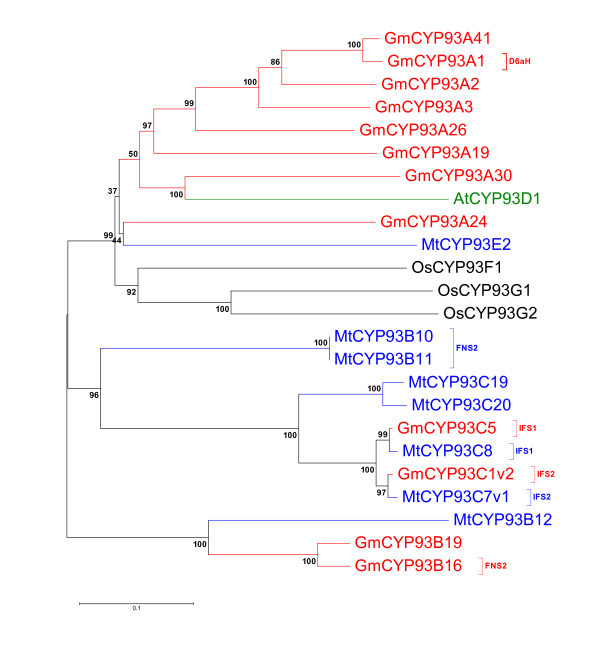
**A phylogenetic tree of CYP93 family proteins. **An unrooted phylogenetic tree of CYP93 family created with amino acid sequences of *Arabidopsis *(Green), soybean (Red), *Medicago *(Blue) and rice (Black). Isoflaovne synthase and flavonoid metabolism known genes were shown in parenthesis. Phylogentic tree was developed using MEGA4.

Flavones are widely distributed in higher plants and isoflavonoids are mainly produced in the leguminous plants. They play significant eco-physiological functions in the adaptation of plants in their biological environments [20]. Flavonoids are synthesized from the phenylpropanoid pathways that utilize several cytochrome P450s. One of the P450 families involved in flavonoid biosynthesis in soybean is the CYP93 family that contains 13 genes compared with 8 genes in *Medicago*, 1 gene in *Arabidopsis *and 3 genes in rice (Figure 1). The CYP93 family includes 9 subfamilies, among which CYP93A encodes dihydroxy-pterocarpan 6α-hydroxylase (D6aH) involved in legume phytoalexin biosynthesis [21]; CYP93B is flavone synthase (FNSII) involved in flavone biosynthesis [22,23]; CYP93C is isoflavone synthase (IFS) involved in isoflavone biosynthesis [24]. *Arabidopsis *CYP93D1 is the only member in the 93D subfamily and it is closely related to soybean CYP93A30, suggesting it can be orthologous to the soybean gene (Figure 1). There are a few sequences in the CYP93F and the CYP93G families, whose functions are still unclear. Sequence similarity indicated that the CYP93 family could have evolved from the more ancient CYP75 by duplication and divergence. The CYP93C family is mostly confined to legumes, whereas the CYP93B sequences have a wider distribution and are found in many species. It is possible that CYP93C might have evolved from the CYP93B subfamily which appears to be a more ancient subfamily [9].

For the non-A type P450s, we selected the CYP74 family for more detailed comparisons. Members of CYP74 family are involved in the formation of plant oxylipins, a large family of metabolites derived from polyunsaturated fatty acids [25]. In *Arabidopsis *genome, only two CYP74s have been identified: CYP74A1 and CYP74B2. *Arabidopsis *CYP74A1 encodes an allene oxide synthase (AOS), which commits 13-hydroperoxy linolenic acid (13-HPOT) to the formation of plant defense hormone, jasmonic acid [26] whereas CYP74B2 is a hydroperoxide lyase (HPL), which converts 13-HPOT to 6-carbon aldehydes and 12-carbon ω-keto-fatty acids [27]. The volatile products from HPL pathway are collectively called green leaf volatiles (GLVs), which can attract the natural enemies of insect herbivores and play an important role in tritrophic interactions. As shown in Figure S1B, in the soybean genome, out of 6 CYP74s, there are 3 CYP74As: CYP74A1, CYP74A21 and CYP74A22. We can assume that these three CYP74As are *bona fide *AOSs. It has been reported that tomato and several other plant species have at least two AOSs leading to the formation of jasmonic acid [25]. Similar to *Arabidopsis*, soybean has only one member of CYP74B; CYP74B15, which is probably a functional HPL. The remaining two soybean CYP74s: CYP74C12 and CYP74C13 do not have homologs in *Arabidopsis*. CYP74Cs from cucumber, melon, almond and rice were shown to be HPLs that have a preference, but not absolute specificity, for 9-hydroperoxides of linoleic and linolenic acids [28-31]. A third AOS gene from tomato was identified, LeAOS3, which is a 9-AOS and a member of CYP74C. So far, 9-AOS has been identified in a few plants including tomato, barley and potato http://metacyc.org/META/new-image?type=PATHWAY&object=PWY-5407[32]. It is quite possible that CYP74C12 and CYP74C13 have activities against 9-HPOT and it would be interesting to identify the products of enzyme reaction to see if there are 9-HPL or 9-AOS.

### P450 gene expression profiling using Affymetrix arrays showed organ-specific and stress-induced expression

To obtain expression profiles of soybean P450 genes, we first utilized the extensive Affymetrix array data publically available at the NCBI database. To identify P450 probe sets on the Affymetrix soybean array (Part #900526), we performed BLASTN searches using the sequences of each probe set against the predicted soybean cDNAs at the TIGR gene index and Phytozome 4.0 genomic sequences. A total of 178 probe sets were found to correspond to P450 genes. Out of these probe sets, 108 represented single genes (at least 100 bp in matching length); the rest matched with more than one gene (55 of them matched with more than two genes). Since multiple targets complicate interpretation of the expression profiles, we focused on the 108 single-target genes for further analysis.

**Figure 2:**
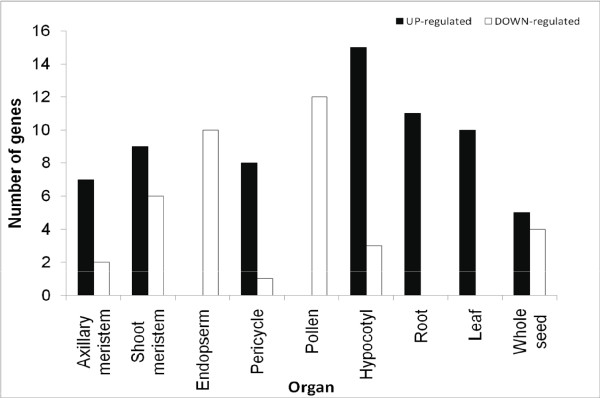
Numbers of up- and down-regulated soybean P450 genes in different organs extracted from Affymetrix soybean microarray dataset.

**Figure 3:**
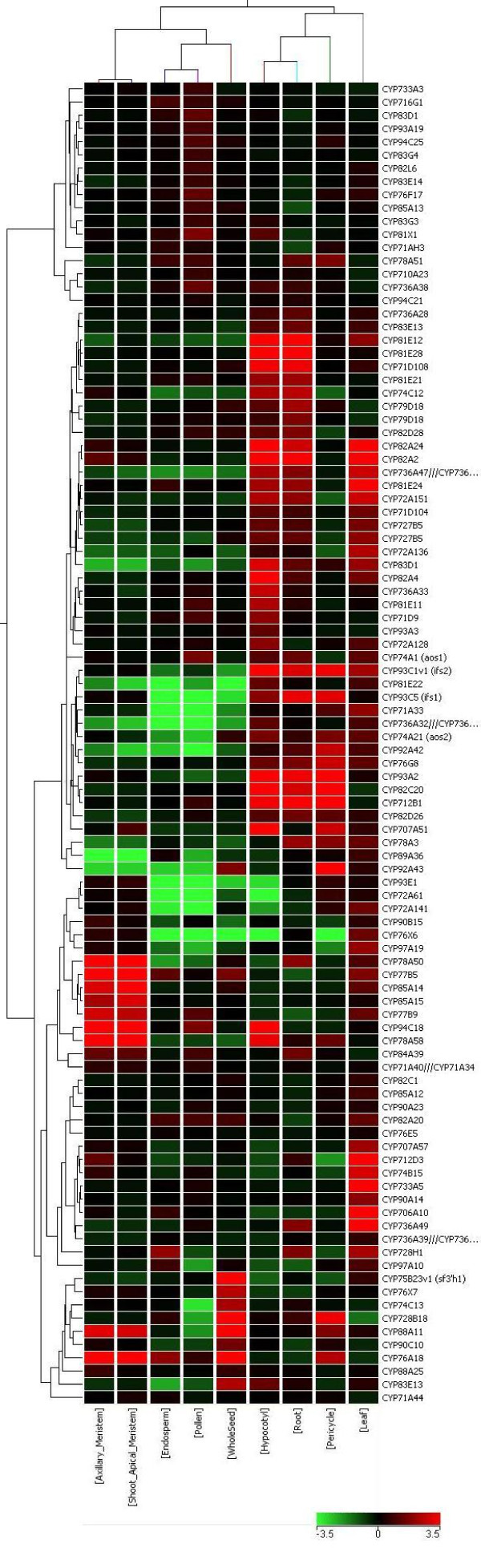
**Expression pattern of soybean P450 genes in different organs according to the analysis of Affymetrix soybean microarray dataset. **The color scale indicates the degree of expression (green: low expression; red: high expression). Heat map was created using GeneSpring 10X.

**Figure 4:**
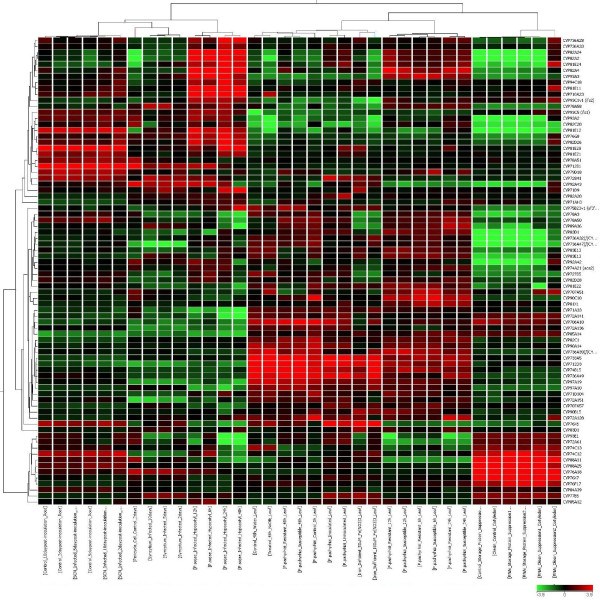
**Expression pattern of soybean P450 genes under stress treatments according to the analysis of Affymetrix soybean microarray dataset. **The color scale indicates the degree of expression (green: low expression; red: high expression). Heat map was created using GeneSpring 10X.

There are 28 microarray libraries that contain organ-specific information in the public database. Following standard microarray data analysis, we identified highly tissue-specific soybean P450 genes (with a cut-off > 3 for up-regulated genes and < −3 for down regulated genes). The data are summarized in Figures 2, 3, 4 and Additional File 3, Table S2. The P450 genes highly induced in leaves, roots, hypocotyl, seeds and axillary meristem are apparent (Figures 2 and 3). For example, out of 108 P450 genes, seven are highly induced and one significantly repressed in the axillary meristem tissues when all genes expressed in all tissues were averaged as control. Each tissue has its own unique set of differentially expressed P450s. Interestingly, *CYP93C1 (IFS1) *and *CYP93C5 (IFS2) *were highly expressed in roots and seeds. More specifically, *IFS1 *is mainly expressed in the root and seed coat; while *IFS2 *is mainly expressed in embryos and pods. These results agree with previous publications using Northern blot analysis, qRT-PCR, and promoter:GUS transgene assays [33].

*CYP93A2*, a member of the 93A subfamily, also showed high expression in roots. *CYP81E12, CYP81E28 *and *CYP81E21 *are also highly expressed in roots. The CYP81E family encodes isoflavone 2'- and 3'-hydroxylases which are involved in isoflavonoid phytoalexin synthesis. Not surprisingly, in *Medicago CYP81E7 *was reported to be highly expressed in roots as well [34]. Other up-regulated and down-regulated P450 genes in each tissue are listed in Additional File 4, Table S3. The functions of these genes are not known, however their strong tissue-specific expression suggests their involvement in those tissue types.

There are 99 microarray libraries in the public database from various stress treatments of soybean. As summarized in Figure 4, the genes *CYP736A28, CYP93E1, CYP82A4, CYP94C18 *and *CYP81E11 *are highly induced in soybean cyst nematode infection. Similarly, *CYP71D9, CYP736A33, CYP72A128, CYP81E28*, and *CYP81E21 *showed strong induction in hypocotyls infected with *Phytophthora Sojae*. Interestingly, *CYP707A51 *and *CYP710A23 *are highly expressed in soybean leaves treated with Nod factor from *B. japonicum *compared to untreated leaves, suggesting they may be involved in the auto-regulation process in which shoots synthesize a mobile signal to suppress additional nodulation in the root after initial rhizobial infection. Under abiotic stress conditions, *CYP71D9 *and *CYP83D1 *were highly expressed under iron deficiency compared to plants grown under sufficient iron. The exact functions of these two genes have not been reported before.

### P450 gene expression profiling using Illumina transcriptome analysis identified organ-specific and rhizobium-induced expression

Given the limitations of the current soybean Affymetrix DNA microarray platform, we also utilized Illumina transcriptome sequencing technology to analyze all 332 soybean P450 gene expressions, using published data from Libault *et al*.[35]. These data sets were generated from total RNAs isolated from nodules, roots, root tips, leaves, flowers, green pods, apical meristem, mock-inoculated and *B. japonicum*-infected root hair cells harvested at 12, 24 and 48 h after inoculation [35]. For comparison, total RNA extracted from stripped roots (i.e. roots devoid of root hairs) were also harvested and sequenced at 48 h after inoculation with *B. japonicum*. Illumina transcriptome analysis allowed a more thorough assessment of all of the 332 soybean P450 genes. Additionally, since Affymetrix array analysis is considered to be less sensitive when comparing different experimental set ups, especially for low abundant RNAs, this deep transcriptome sequence analysis allowed a clear distinction between low-abundance and undetectable transcripts.

**Figure 5:**
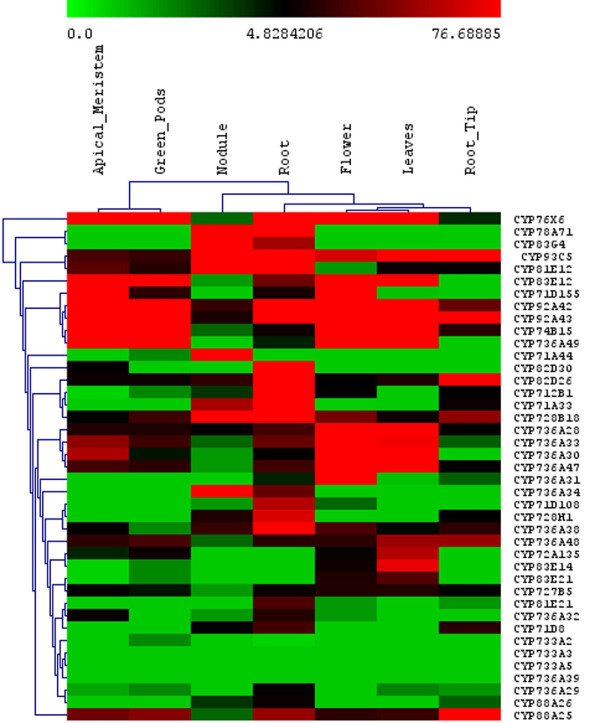
**Expression pattern of soybean P450 genes in different organs according to the analysis of Illumina transcriptome dataset. **The color scale indicates the degree of expression (green: low expression; red: high expression). Heat map was created using GeneSpring 10X.

**Figure 6:**
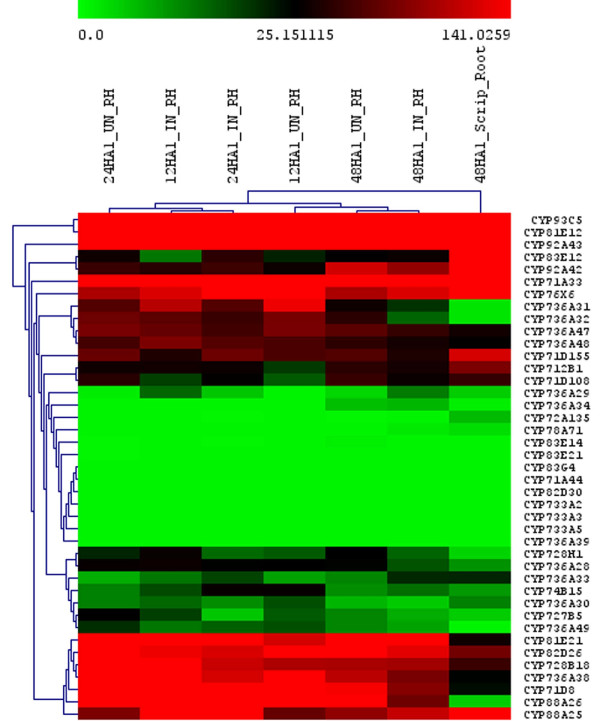
**Expression of soybean P450 genes in root hair infection process according to the analysis of Illumina transcriptome dataset. **The labels are 24HA1_UN_RH, root hair sample 24 hours after mock treatment; 12HA1_IN_RH, root hair sample 12 hours after infection; 24HA1_IN_RH, root hair sample 24 hours after infection; 12HA1_UN_RH, root hair sample 12 hours after mock treatment; 48HA1_UN_RH, root hair sample 48 hours after mock treatment 48HA1_IN_RH, root hair sample 48 hours after infection; 48HA1_Script_Root, stripped root sample 48 hours after infection.

From these Illumina data, the P450 genes highly expressed in different tissue were hierarchically clustered using the MeV v4.5.1 software. The entire expression profile in all tissues and treatments is shown in Additional File 5, Figure S2. To simplify the figure, the expression of P450s involved in isoflavone biosynthesis, CYP93 family genes and P450 families present only in soybean and not in *Arabidopsis*, including CYP92, CYP733, CYP735 and CYP736 have been highlighted (Figures 5 and 6). In Figure 5, very different expression profiles in apical meristem, green pods, nodules, roots, flowers, leaves, and root tips can be seen among selected P450s. For example, CYP733A members were not detected in any of the tissues, suggesting a more narrow distribution beyond our collection of cDNA libraries. In Figure 6, root hairs and stripped roots were profiled with or without rhizobia induction. Isoflavone biosynthetic enzymes were expressed in all tissues and treatments, in contrast with CYP733A and CYP83 family where no expression could be detected in any tissues. Interestingly, *CYP88A3 *appeared to be expressed only in the root hairs, not in the roots stripped of root hairs. Similarly, *CYP736A31 *and *CYP736A32 *exhibit much higher expression in the root hairs than in the stripped roots. In contrast, the same family *CYP736A47 *and *CYP736A48 *had an opposite expression pattern, with significantly higher expression in roots than in root hairs.

Of all P450 genes analyzed, the ones highly expressed in nodules, roots, leaves and flowers are summarized in Table 3. The most abundant P450 genes in each tissue, as shown by the calculated tag sequence numbers, are *CYP83G4 *(nodules), *CYP76X6 *(apical meristem, green pods and leaves), *CYP83E12 *(flowers), and *CYP93C5 *(roots and root tips). Clearly, *CYP76X6 *is the most abundant P450 in green tissues (function unknown); while the isoflavone synthetic enzyme IFS is the major P450 in roots.

**Table 3:** Organ specific expression of P450 genes using Illumina transcriptome.

	**Nodule**	**Apical Meristem**	**Flower**	**Green Pods**	**Leaf**	**Root**	**Root Tips**
	
CYP78A71	**7763.1**	0	0	0	0	**102.7**	0
CYP83G4	**4737.8**	0	0	0	0	50.1	0
CYP71A44	**524.1**	0	0	2.2	0	0	0
CYP736A34	**95.2**	0	0	0	0	34.0	0
CYP93C5	**889.5**	25.0	65.7	20.1	159.2	**3334.8**	**2596.7**
CYP81E12	**404.8**	30.0	1.9	15.6	8.1	**2962.0**	7.1
CYP82D26	18.3	9.2	4.8	8.9	13.9	**310.7**	139.4
CYP712B1	3.9	0.8	4.8	2.2	0	**231.8**	6.1
CYP71A33	51.9	0	1.0	0	0	**172.3**	9.1
CYP71D108	1.9	0	2.9	0	0	**56.0**	0
CYP72A135	0	4.2	7.7	8.9	55.8	0	0
CYP83E21	0	0.8	13.5	2.2	27.9	0	0
CYP83E12	1.9	**512.4**	**1403.1**	**967.1**	**145.2**	36.5	0
CYP74B15	2.9	**136.6**	**468.4**	**113.9**	**560.1**	9.3	16.2
CYP71D155	0	**691.6**	**89.8**	**17.9**	**1.1**	11.0	0
CYP76X6	2.9	**8328.7**	**1044.9**	**12422.1**	**1119.0**	188.5	4.0

List of P450 genes and their expression intensity values in different organs. Value zero indicates no expression.

We were interested in the rhizobium-induced P450 genes. Out of 12 genes in the CYP736 family, *CYP736A34 *showed high expression in nodules and roots (Table 3 and Figure 6). *CYP78A71, CYP83G4 *and *CYP71A44 *were highly expressed in nodules. Almost no expression was detected in other tissues. Considering there are only two members of the CYP83 family in *Arabidopsis*, but five members in *M. truncatula *and six members in *Lotus japonicus*, the CYP83 family may play important roles in legumes, although its function remains unknown [16].

**Figure 7:**
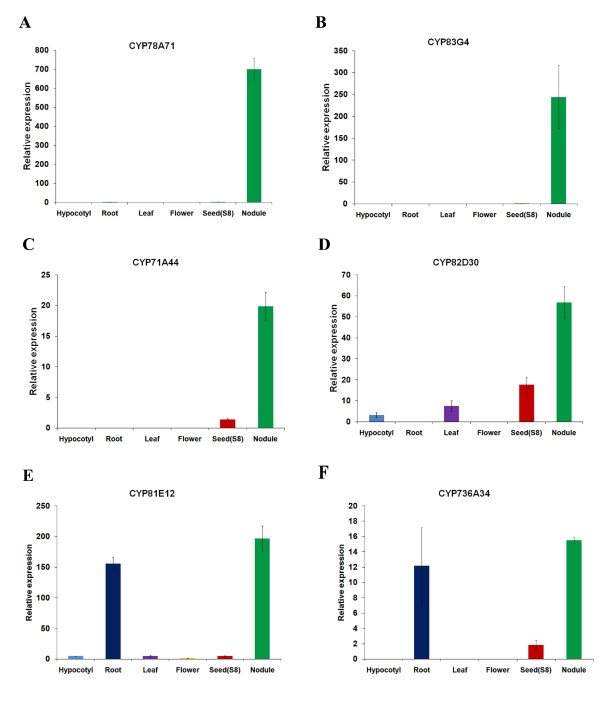
**qRT-PCR confirmation of soybean P450 genes highly expressed in root and nodule as revealed by the analysis of Microarray and Solexa transcriptome datasets. **Error bars represent SE (Standard error) of three replications.

To confirm these findings from Illumina sequencing, we carried out qRT-PCR analysis on the four nodule-specific genes and a set of selected other P450 genes. Total RNAs were isolated from hypocotyls, roots, leaves, flowers, seed (stage R8), and dissected nodules. The expression levels of the above-mentioned *CYP78A71, CYP83G4, CYP71A44*, and *CYP82D30 *were measured. All four genes showed dominant nodule-specific expression, consistent with the Illumina data. Two additional genes, *CYP81E12 *and *CYP736A34*, showed strong root and nodule expression, also fitting the Illumina data very well. All six genes on Figure 7 had lower expression in other tissues.

**Figure 8:**
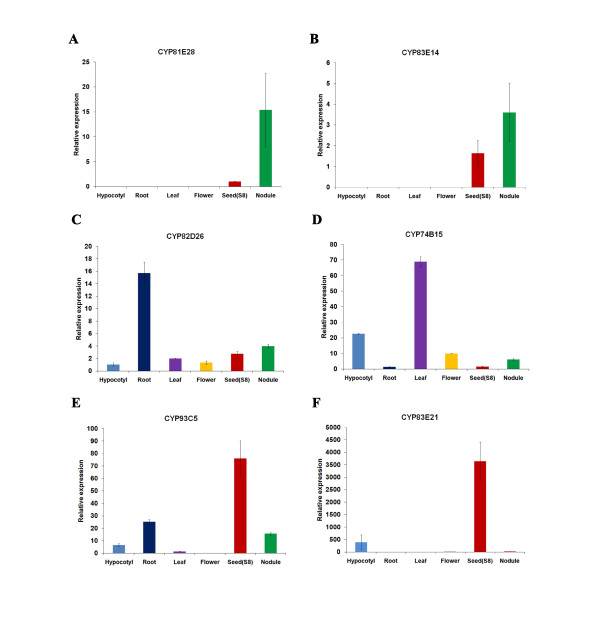
**qRT-PCR confirmation of soybean P450 gene expression in different organs as revealed by the analysis of Microarray and Solexa transcriptome datasets. **Error bars represent SE of three replications.

As controls, *CYP82D26, CYP74B15, CYP93C5*, and *CYP83E21 *genes were also analyzed by qRT-PCR (Figure 8). All four genes showed exactly the same expression pattern as suggested by Illumina analysis. *CYP93C5 (IFS1*) has been characterized by other expression analysis methods previously and all data are consistent with our qRT-PCR analysis. Taken together, we have discovered four major nodulation-specific P450 genes, which will be functionally characterized in the future.

We also analyzed another set of Illumina data recently published by Severin *et al*. [36]. Since the normalization methods were different in these two data sets, we were unable to combine these two transcriptome data. However, the expression profiles of highlighted genes in the comparable tissue types were similar in these two data sets (see Additional File 6, Table S4). For example, the above-mentioned *CYP78A71, CYP83G4*, and *CYP71A44 *all showed nodule-specific expression while *CYP82D30 *expressed in roots and nodules.

### Co-expression analysis revealed co-ordinately expressed metabolic enzymes of some P450s

Co-expression analysis assumes that some genes of the same biochemical pathway are co-ordinately regulated at the transcriptional level. In general, P450s catalyze slow and irreversible steps in many branches of the plant metabolic pathways. They have been shown to be co-expressed and regulated with non-P450 genes with known functions in the same pathway [37]. For example, the functions of several uncharacterized P450 genes in Arabidopsis were predicted using co-expression analysis and confirmed experimentally later [11,38]. In this study, we performed co-expression analysis comparing the expression of each P450 with that of genes present in the Affymetrix Soybean gene chip.

The annotation information of the current Affymetrix soybean gene chip was combined with expression data to identify genes and pathways co-expressed with the P450 genes. For each P450 gene, we calculated Pearson correlation coefficients (r-value) with genes present on the Affymetrix soybean array. Only the genes with significant coefficients (r > 0.7) were selected for pathway analysis. We first investigated a known soybean P450 gene to test our co-expression analysis approach. As shown in Table 4, *CYP93C5 *isoflavone synthase (IFS1) gene co-expresses strongly with several isoflavonoid-related metabolic enzymes, including chalcone isomerase 1B1 (r = 0.91), and chalcone isomerase 1B2 (r = 0.87). Interestingly, the few transcription factors, such as Myb76 and bZIP42 that are co-expressed with these genes could be the missing transcription factors that regulate isoflavone biosynthesis under stress conditions. Functional annotation of *Arabidopsis *and soybean are shown in Table 5.

**Table 4:** Co-expression analysis of selected soybean P450 genes.

Soybean Gene	Co-expressed genes	r value
**CYP93C5-IFS1**		
	**Isoflavone synthase 1**	**1**
	Chalcone isomerase 1B2	0.91
	Chalcone isomerase 1B1	0.87
	Peroxidase, pathogen-induced	0.85
	Copper amino oxidase	0.82
	NAC domain protein	0.81
	Peroxidase (PC7)	0.80
	Peroxidase precursor (GMIPER1)	0.79
	MYB transcription factor MYB67	0.79
	Chalcone isomerase 2	0.79
	NORK protein	0.79
	KNT1	0.78
	Transcription factor bZIP42	0.78
	Plastid glucose-6-phosphate	0.77
	Kunitz trypsin inhibitor p20-1-like protein	0.77
	Dof3	0.76
	Chalcone isomerase	0.76
	Glutathione S-transferase GST 17	0.75
		
**CYP728H1**		
	**CYP728H1**	**1**
	2'-hydroxy isoflavone/dihydroflavonol reductase homolog	0.92
	4-coumarate:CoA ligase isoenzyme 2	0.90
	Cytochrome P450 (CYP98A2)	0.86
	RecA/Rad51/DMC1-like protein	0.83
	Peroxidase	0.81
	Endo-xyloglucan transferase	0.80
	bZIP transcription factor bZIP17	0.79
	Essex desiccation protectant protein Lea14 homolog	0.79
	NAC domain protein (NAC18)	0.79
	WD-repeat cell cycle regulatory protein	0.79
	4-coumarate:coenzyme A ligase	0.79
	31 kDa protein	0.78
	Transcription factor bZIP18	0.77
	Hydroxyproline-rich glycoprotein (sbHRGP2) mRNA, 3' end	0.76
	Early nodulin	0.76
	Protease inhibitor	0.76
	Wee1	0.76
		
**CYP736A32/34**		
	**CYP736A32//CYP736A34**	**1**
	Lectin	0.93
	SOS2-like protein kinase	0.92
	Cultivar Wenfeng7 purple acid phosphatase-like protein (Pap3)	0.91
	Lipoxygenase L-5	0.88
	Cysteine proteinase	0.88
	Peroxisomal ascorbate peroxidase	0.88
	Thiol protease isoform B	0.86
	Phosphoglycerate mutase-like protein	0.86
	Coronatine-insensitive 1	0.84
	Catalase (cat4)	0.83
	Sorbitol-like transporter	0.83
	MYB transcription factor MYB48	0.82
	Phosphoenolpyruvate carboxylase	0.82
	Indole-3-acetic acid induced protein ARG-2 homolog	0.81
	Lipoxygenase (lox7)	0.79
	WRKY39 protein	0.77
	Glutathione S-transferase GST 8	0.77
	Mitogen-activated protein kinase 1 (MAPK1)	0.76
	Ribulose-1,5-bisphosphate carboxylase small subunit	0.76
	Clone pSXET1a xyloglucan endotransglycosylase precursor (XET1)	0.76
	SOC1	0.76
	OAS-TL3 cysteine synthase	0.76
	WRKY23 (WRKY23)	0.76
	Nitrate reductase (NiR)	0.75
	NAC domain protein	0.75
	bZIP transcription factor bZIP105	0.74
	Allene oxide synthase (AOS2)	0.74
	Chalcone synthase 1	0.72
	CYP75B23v1 (sf3'h1)	0.70

Selected P450 genes and their co-expressed genes are listed with Pearson correlation coefficient (r value >0.7)

**Table 5:** List of *Arabidopsis *and soybean P450 genes and orthologues with known functions.

AGI locus	Arabidopsis Gene	Soybean orthologue	Percentage Identity	Function in Arabidopsis	Reference -Arabidopsis function
At1g11680	CYP51A2	CYP51G1	80.9	Obtusifoliol 14α-demethylase	(Kushiro et al. 2001); (Kim et al. 2005b)
At2g30770	CYP71A13	CYP71A9	37.9	Conversion of indole-3-acetaldoxime, camalexin biosynthesis	(Nafisi et al. 2007)
At3g26830	CYP71B15	CYP83E8		Conversion of s-dihydrocamalexic acid to camalexin	(Bottcher et al. 2009); (Zhou et al. 1999)
At1g17060	CYP72C1	CYP72A69	44.3	Exact substrate not identified	(Nakamura et al. 2005); (Takahashi et al. 2005)
At2g30490	CYP73A5	CYP73A11	84.4	Cinnamic acid 4-hydroxylase (*t*-CAH)	(Mizutani et al. 1997)
At5g42650	CYP74A	CYP74A1	56.4	Allene oxide synthase (AOS)JA	(Laudert et al. 1996); (Park et al. 2002)
At4g15440	CYP74B2	CYP74B15	60.2	Hydroperoxide lyase (HPL) JA	(Bate et al. 1998)
At5g07990	CYP75B1	CYP75B43	52.6	3′-hydroxylase for narigenin, dihydrokaempferol (F3′H)	(Schoenbohm et al. 2000)
At5g04630	CYP77A4	CYP77A3/A12	68.1	Catalyze the formation of three mono-epoxides of alpha-linolenic acid	(Sauveplane et al. 2009)
At3g10570	CYP77A6	CYP77A12/A12	65	Chain hydroxylase - for cutin synthesis for morphology of flower	(Li-Beisson et al. 2009)
At1g13710	CYP78A5	CYP78A72	62.8	KLU control organ size, control leaf growth	(Wang et al. 2008); (Anastasiou et al. 2007)
At5g05260	CYP79A2	CYP79D17	49.1	Conversion of phenylalanine to oxime	(Whittstock and Halkier 2000)
At4g39950	CYP79B2	CYP79D21	51.1	Conversion of tryptophan, tryptophan analogs to oxime	(Hull et al. 2000); (Mikkelsen et al. 2000)
At2g22330	CYP79B3	CYP79D21	50.9	Conversion of tryptophan to oxime	(Hull et al. 2000)
At1g16410	CYP79F1	CYP79D17/21	40.7	Mono to hexahomomethionine in synthesis of aliphatic glucosinolates	(Hansen et al. 2001); (Reintanz et al. 2001); (Chen et al. 2003)
At1g16400	CYP79F2	CYP79D17/22	40.2	Long chain penta and hexahomomethionine in synthesis of long chain aliphatic glucosinolates	(Reintanz et al. 2001); (Chen et al. 2003)
At5g57220	CYP81F2	CYP82A3	47.6	Conversion of indole-3-yl-methyl to 4-hydroxy-indole-3-yl-methyl glucosinolate,	(Bednarek et al. 2009); (Pfalz et al. 2009)
At4g13770	CYP83A1	CYP83E8/CYP736A29	37	Oxidation of methionine-derived oximes;	(Bak and Feyereisen 2001); (Naur et al. 2003)
At4g31500	CYP83B1	CYP83E8/CYP736A30	45	Oxidation of indole-3-acetaldoxime	(Bak et al. 2001); (Naur et al. 2003)
At4g36220	CYP84A1	CYP8438/39/21	73.4	5-hydroxylase for coniferaldehyde, coniferyl alcohol and ferulic acid (F5H)	(Ruegger et al. 1999); (Humphreys et al. 1999)
At5g38970	CYP85A1	CYP85A12	68.9	C6-oxidase for 6-deoxycastasterone, other steroids	(Shimada et al. 2001); (Shimada et al. 2003)
At3g30180	CYP85A2	CYP85A13	70.3	C6-oxidase for 6-deoxycastasterone, other steroids;Conversion of castasterone to brassinolide	(Shimada et al. 2003); (Nomura et al. 2005)
At5g58860	CYP86A1	CYP86A37	72.8	ω-hydroxylase for satur. and unsat. C12 to C18 fatty acids	(Benveniste et al. 1998)
At4g00360	CYP86A2	CYP86A66	71.2	ω-hydroxylase for satur. and unsat. C12 to C18 fatty acids	(Duan and Schuler 2005); (Xiao et al. 2004)
At2g45970	CYP86A8	CYP86A66/67	76.4	ω-hydroxylase for satur. and unsatur. C12 to C18 fatty acids	(Wellesen et al. 2001)
At5g23190	CYP86B1	CYP86B9/10/11	69.5	C22 and C24 fatty acids, accumulated in the suberin polyester.	(Compagnon et al. 2009)
At1g05160	CYP88A3	CYP88A11/25	63.5	Multifunctional *ent*-kaurenoic acid oxidase	(Helliwell et al. 2001)
At2g32440	CYP88A4	CYP88A26	53.2	Multifunctional *ent*-kaurenoic acid oxidase	(Helliwell et al. 2001)
At5g05690	CYP90A1	CYP90A14/23/24	75.8	23α-hydroxylase for 6-oxo-cathasterone	(Szekeres et al. 1996)
At3g50660	CYP90B1	CYP90B15/18/17	71.3	22α-hydroxylase for 6-oxo-campestanol, campesterol and cholesterol	(Choe et al. 1998); (Fujita et al. 2006)
At4g36380	CYP90C1	CYP90C8/9/10	55.3	Conversion of typhasterol to castasterone, C-23 hydroxylation	(Ohinishi et al.); (Kim et al. 2005a)
At3g13730	CYP90D1	CYP90D12/13	61.9	Exact substrate in downstream BR synthesis not identified	(Kim et al. 2005a); (Greer et al. 2007); (Ohinishi et al. 2006)
At1g57750	CYP96A15	CYP94B13	42.4	Formation of secondary alcohols and ketones in cuticular wax of stem, acyl CoA reductase	(Greer et al. 2007)
At1g31800	CYP97A3	CYP97A10/19	70.7	β −ring hydroxylase on carotenes	(Kim and DellaPenna 2006)
At3g53130	CYP97C1	CYP97A16/17	79	ε −ring hydroxylase on carotenes	(Tian et al. 2004)
At2g40890	CYP98A3	CYP98A2/47	80	3′-hydroxylase for *p*-coumaryl shikimic/quinic acids (C3′H)	(Schoch et al. 2001); (Kai et al. 2006)
At5g25900	CYP701A3	CYP701A25/16	61.2	Multifunctional *ent*-kaurene oxidase	(Helliwell et al. 1998)
At1g01280	CYP703A2	CYP703A8	74.9	Sporopollenin synthesis, pollen development	(Morant et al. 2007)
At1g69500	CYP704B1	CYP704B28	75.5	Fatty acid -sporopollenin biosynthesis -pollen	(Dobritsa et al. 2009)
At4g19230	CYP707A1	CYP707A16	68.4	8′-hydroxylase for ABA inactivation	(Saito et al. 2004); (Kushiro et al. 2004)
At2g29090	CYP707A2	CYP707A45	61.9	8′-hydroxylase for ABA inactivation, Enhancement of ABA catabolism	(Saito et al. 2004); (Kushiro et al. 2004)
At5g45340	CYP707A3	CYP707A16/56	71.6	8′-hydroxylase for ABA inactivation	(Saito et al. 2004); (Kushiro et al. 2004)
At3g19270	CYP707A4	CYP707A53/59	65.1	8′-hydroxylase for ABA inactivation	(Saito et al. 2004); (Kushiro et al. 2004)
At2g34500	CYP710A1	CYP710A22/23	66.3	C-22 desaturase for β-sitosterol	(Morikawa et al. 2006)
At2g34490	CYP710A2	CYP710A22/24	62	C-22 desaturase on 24-*epi*-campesterol and β-sitosterol	(Morikawa et al. 2006)
At2g26170	CYP711A1	CYP711A23/24/25/26	70.6	Caretnoid,core phenylpropanoid metabolism	(Booker et al. 2005)
At2g26710	CYP734A1	CYP734A17/20/21	76.9	26-hydroxylase for brassinolide and castasterone	(Neff et al. 1999)
					
	
	**Soybean gene**	**Arabidopsis orthologue**	**Percentage identity**	**Function in soybean**	**Reference-Soybean function**
	
	CYP93C1	CYP93A41	40.9	Isoflavone synthase (IFS1)	(Jung et al. 2000)
	CYP93C5	CYP93A41	40.3	Isoflavone synthase (IFS2)	(Jung et al. 2000)
	CYP71D09	CYP71B34	39.1	Flavonoid 6-hydroxylase	(Latunde-Dada et al. 2001)
	CYP73A11	CYP73A5	84.4	Cinnamate 4-hydroxylase	(Schopfer et al. 1998)
	CYP93A1			Dihydroxypterocarpan 6a-hydroxylase (D6aH)	(Schopfer and Ebel 1998); (Schopfer et al. 1998)
	CYP71A10			Metabolism of phenylurea herbicides	(Siminszky et al. 1999)

We then performed the co-expression analysis on many of the 108 P450 genes on the Affymetrix arrays (Table 4 and Additional File 6, Table S4). We selected three examples and highlight them in Table 4. The *CYP728H1 *showed high expression in roots and nodules, and was co-expressed with 4-coumarate:CoA ligase isoenzyme 2, cytochrome P450 98A2 and 4- coumarate:coenzyme A ligase genes which are involved in phenylpropanoid metabolism. *CYP728H1 *is also co-expressed with endo-xyloglucan transferase, NAC transcription factor, 2'-hydroxy isoflavone and early nodulin genes. Gene expression and co-expression analysis suggests that *CYP728H1 *may play an important role in isoflavone metabolism, as well as during root and nodule development.

The CYP736 family is present in soybean but not in *Arabidopsis*. *CYP736A34 *showed high expression in roots and nodules (Figure 7). Co-expression analysis showed that the expression of this gene is highly correlated with lipoxygenase, lectin and *CYP83D1*, all of which are involved in root and nodule development (Table 4). Some of the co-expressed genes are defense related genes such as cysteine proteinase. Since defense response is one of the early nodulation events, *CYP736A34 *may be functioning at the early stages of this symbiotic process.

These analyses show that co-expression analysis combined with pathway mapping of co-expressed genes is a powerful tool to identify genes encoding enzymes acting in the same biochemical pathway or biological process. Unfortunately, annotation of soybean genes in the Affymetrix gene chip is not as comprehensive as other model species. Many of the co-expressed genes have unknown or un-confirmed functions. A majority of the P450s cannot be mapped to specific pathways. However, this approach still provides important leads to large sets of uncharacterized soybean P450s, and with improved annotation of soybean genes in the near future, we should be able to extract more functional information of soybean P450s.

## Conclusions

Soybean is an important leguminous crop. With the advent of soybean genome sequencing, it is possible to study large gene families of soybean. We targeted one of the most challenging families in plants, the cytochrome P450 genes, and investigated their genetic make-up, gene distributions, expression profiles, and co-expressed associates. Cytochrome P450s are indispensable for soybean growth, development, and defense against pathogens. They may play important roles for soybean symbiotic interaction with rhizobacteria. Overall, we identified 332 full-length P450 genes and 378 pseudogenes in the genome. We used publicly available microarray libraries and identified few tissue-specific and stress responsive soybean P450s. The tissue-specific expression patterns of some P450 genes were confirmed by qRT-PCR. In addition, the expressions patterns of all 332 soybean P450 genes were obtained through the analysis of Illumina transcriptome datasets. The co-expression analysis on some of the P450 genes was performed using the Affymetrix array datasets. We demonstrate that gene co-expression analysis is a useful tool to guide our further study on the function of uncharacterized genes. Importantly, the identification of nodule-specific P450s and their further exploitation may help us to uncover the intriguing process of soybean and rhizobium interaction.

## Methods

### Identification of soybean cytochrome P450 genes

Existing soybean P450 genes and corresponding *Glyma *numbers (location markers in soybean genome) were retrieved from the Cytochrome P450 Homepage website http://drnelson.uthsc.edu/CytochromeP450.html. Additional full-length soybean genes were identified based on the cytochrome P450 domain predictions from Pfam [39], Panther [40], and KOG [41] separately. The search results were consolidated with existing soybean P450s. From these sequences, the pseudogenes were identified using existing criteria listed on the P450 homepage website.

### Computational phylogenetic analysis

Protein sequences of soybean P450 genes were obtained based on Phytozome 4.0. For comparison, a collection of P450s from *Arabidopsis thaliana *(245 genes) and the corresponding CYP names were retrieved from *Arabidopsis *cytochrome P450 web-based resource http://www.p450.kvl.dk/p450.shtml[42]. In addition, CYP93 family proteins from *Medicago truncatula *and rice were selected from Genbank for relationship analysis across species. Multiple sequence alignment were performed using the BLOSUM matrix (Gap opening and extension penalties of 25 and 1, respectively), using the ClustalW algorithm-based AlignX module from the Mega4 software [18]. The phylogenetic tree was constructed using the Neighbour-Joining Tree method by P-distance in MEGA4. The significance level of the neighbor-joining analysis was examined by bootstrap testing with 1000 repeats.

### 1.3 Soybean Affymetrix array analysis

Publically available experiments and arrays were listed at the NCBI GEO and Array Express database http://www.ncbi.nlm.nih.gov/sites/entrez. For tissue-specific expression, six experiments (28 arrays) were analyzed using RNAs isolated from different tissues and developmental stages. For stress-induced experiments, eight experiments (99 arrays) were analyzed using RNAs isolated from various abiotic and biotic stress treatments. Expression analysis was carried out using GeneSpring 10GX (Agilent Technologies, Forster City, CA). First, all microarray data were normalized and summarized using the RMA algorithm. Signals from each probe set were then normalized to the median of their values across the entire dataset. Quality control of the dataset was performed using Principal Components Analysis (PCA) to confirm that there were no outlying replicate samples, and dye labeling had no associated bias. Data were combined from replicate samples and grouped in experiment interpretations. Gene list (CYP genes) were generated by expression with cut off P < 0.05. Statistical analysis was performed using One-Way ANOVA with Posthoc-TukeyHSD test to determine statistical significant difference between means. Signal intensities from organ and tissue samples were then compared to the average signal intensities during normalization. In stress experiments, signal intensities from treatment groups were compared to signal intensities from the corresponding control samples to generate fold changes. Genes that were significantly up or down regulated (>two fold) were selected for hierarchical clustering. Hierarchical clustering tree was created based on Pearson correlation coefficient under each experimental condition. Co-expression analysis and pathway mapping were performed in GeneSpring as well. The selected P450 genes were mean-centered and Pearson correlation coefficients (r-values) calculated between each P450 probe set. Co-expressed genes with r > 0.7 were retrieved and the corresponding available biochemical pathways were extracted from the Plant Metabolic Network http://www.plantcyc.org/[43].

### 1.4 Soybean Illumina expression data analysis

Solexa sequencing libraries for fourteen different conditions including nodules, roots, root tips, leaves, flowers, green pods, apical meristem, mock-inoculated and *B. japonicum*-infected root hair cells harvested at 12, 24 and 48 h after inoculation, generated and analyzed by Libault *et al*. [35,44] were utilized to quantify the transcriptomics expression of soybean genes (i.e. the number of sequence reads/million reads aligned). Read counts used in expression analyses were based on the subset of uniquely aligned reads that also overlapped the genomic spans of the Glyma1 gene predictions. Read counts for a given sample were normalized by using values for a gene's uniquely aligned read counts per million reads uniquely aligning within that sample. A total of 51,529 annotated soybean genes (74.5% of the 69,145 putative, annotated soybean genes) were found to be expressed in at least one condition.

### 1.5 Plant materials, growing conditions, and RNA extraction

Soybean (*G. max *L. cv. Jack) seeds were germinated in three-gallon pots containing Promix (Home Depot, Atlanta, GA). The seedlings were grown in Conviron growth chamber (26/20 °C day/night temperature, photoperiod of 14/10 h, 800 μmol m^-2 ^s-^1 ^light intensity and 60% humidity). When the seedlings developed four nodes and three fully opened trifoliate leaves, approximately 25 d after sowing, the roots, hypocotyls, leaves were collected. Flowers and early R8 stage seeds were harvested 55 and 100 d after sowing, respectively. Collected tissues were immediately frozen in liquid nitrogen. Soybean plants inoculated with *B. japonicum *USDA110 strain was used for nodulation as described previously [45].

Total RNA was isolated from plants using TRIZOL reagent (Invitrogen, Carlsbad, CA). For each sample, 10 μg of total RNA were digested with RNase-free DNaseI (Promega, Madison, WI) to remove any genomic DNA contamination. After DNaseI treatment, RNA concentration was determined again using a NanoDrop ND-1000 UV-Vis spectrophotometer (NanoDrop Technologies, Wilmington, DE). First-strand cDNA was synthesized from 2 μg total RNA using the Superscript III first strand synthesis system (Invitrogen). All cDNA samples were diluted 50-fold in sterile water for real time PCR reaction.

### 1.6 Quantitative RT-PCR analysis

Gene specific primers were designed using ProbeFinder Version 2.44 https://www.roche-applied-science.com. The list of genes and primers used for amplification are shown in Additional File 7, Table S5. Primer specificity was further confirmed by blasting each primer sequence against Phytozome http://www.phytozome.net/search.php?show=blast using the BLASTN algorithm. Soybean *Actin *and *Ubiquitin *genes were used as internal controls for gene expression studies [46]. Quantitative RT-PCR (qRT-PCR) reactions were performed in 96-well plates (StepOne Plus Real Time PCR System; Applied Biosystems, Foster City, CA) for all tissues tested. Clontech's SYBR Advantage qPCR Premix was used for the qRT-PCR reactions. Primer sets (0.2 μM final concentrations for each primer) were used in a final volume of 10 μL per well. The thermal profile of the qRT-PCR reactions was 95°C for 5 min, followed by 40 cycles of 95°C for 15 sec, and 60°C for 10 sec and 72°C for 20 sec. Melting curve of each PCR amplicon was obtained by adding the following cycling condition: 95°C for 15 sec followed by a constant increase of the temperature between 60 to 95°C at an increment of 0.3°C/cycle.

## Authors' contributions

SKG, OY designed all the experiments. SKG performed Phylogentic tree construction, gene expression and co-expression analysis. JT carried out the computational and bioinformatics analysis. SKG, NB and SP designed qRT-PCR experiments and data analysis. NB performed all the qRT-PCR analysis. HC involved in design and co-expression analysis. OY, DX conceived of the project. YCA provided help on GeneSpring software. All authors read and approved the final manuscript.

## Supplementary Material

Additional file 1 **Table S1 Comparison of P450 families among soybean, *Medicago*, *Arabidopsis*, rice, poplar, grape and moss**. A. List of A-type P450 families. B. List of Non-Atype P450 families. For each family number of genes and pseudogenes were compared among different plant species. Click here for file

Additional file 2 **Figure S1 Phylogenetic tree of all soybean P450s**. S1A. A-type P450s of soybean and *Arabidopsis*. S1B. Non-A type P450s of soybean and *Arabidopsis*. Soybean P450s are shown in blue and *Arabidopsis *P450s are shown in green. Trees were constructed using MEGA4. Click here for file

Additional file 3 Table S2 Organ-specific expressions of soybean P450 genes based on Affymetrix arrays. Click here for file

Additional file 4 **Table S3 Organ specific expression of P450 genes using Illumina transcriptome**. List of P450 genes and their expression intensity values in different organs. Value zero indicates no expression. Click here for file

Additional file 5 **Figure S2 Gene expression of 332 soybean P450 genes according to the analysis of Illumina transcriptome dataset**. Gene expression in different organ and root hair inoculation process. Click here for file

Additional file 6 Table S4 Co-expression analysis of soybean P450 genes based on Affymetrix arrays. Click here for file

Additional file 7 Table S5 List of primer pairs used in qRT-PCR Click here for file
