# Supplementary material for: Whole genome co-expression analysis of soybean cytochrome P450 genes identifies nodulation-specific P450 monooxygenases
Source: BMC Plant Biol. 2010 Nov 9;10:243. doi: 10.1186/1471-2229-10-243 (PMC3095325; doi:10.1186/1471-2229-10-243)
Supplement: Additional file 1 — Table S1 Comparison of P450 families among soybean, Medicago, Arabidopsis, rice, poplar, grape and moss. A. List of A-type P450 families. B. List of Non-Atype P450 families. For each family number of genes and pseudogenes were compared among different plant species. [file 1471-2229-10-243-S1.PDF]

Table S1A. List of A-type P450 families. For each family genes and pseudogenes were compared among different plant species

Family	Soybean		Medicago		Arabidopsis		Rice	
	Genes	Psuedogenes	Genes	Psuedogenes	Genes	Psuedogenes	Genes	Psuedogenes
**A-TYPE**								
**CYP71 clan**								
CYP71	55	65	37	22	52	2	84	28
CYP73	3	2	1	0	1	0	3	1
CYP75	7	5	0	0	1	0	3	0
CYP76	14	29	6	0	8	1	29	12
CYP77	4	0	2	0	5	2	2	0
CYP78	11	8	1	0	6	0	8	0
CYP79	5	4	3	0	7	5	4	0
CYP80	0	0	0	0	0	0	0	0
CYP81	12	19	5	0	18	0	12	1
CYP82	24	9	10	1	5	0	0	0
CYP83	12	11	9	0	1	0	0	0
CYP84	3	3	3	0	2	0	3	1
CYP89	8	4	9	0	7	0	14	6
**CYP92**	**2**	**0**	**1**	**0**	**0**	**0**	**9**	**5**
CYP93	13	22	8	0	1	0	3	4
CYP98	2	1	1	0	3	0	2	2
CYP99	0	0	0	0	0	0	2	0
CYP701	2	0	1	0	1	0	5	0
CYP703	1	1	1	0	1	0	1	0
**CYP705**	**0**	**0**	**0**	**0**	**26**	**8**	**0**	**0**
CYP706	3	4	1	0	7	0	4	0
CYP712	2	0	1	0	2	0	0	0
CYP723	0	0	0	0	0	0	2	1
CYP726	0	0	0	0	0	0	0	0
**CYP736**	**12**	**15**	**1**	**0**	**0**	**0**	**0**	**0**
**Total**	**195**	**202**	**101**	**23**	**154**	**18**	**190**	**61**

Table S1B. List of Non A-type P450 families. For each family genes and pseudogenes were compared among different plant species

Family	Soybean		Medicago		Arabidopsis		Rice	
	Genes	Psuedogenes	Genes	Psuedogenes	Genes	Psuedogenes	Genes	Psuedogenes
**NON-A**								
**CYP51 clan**								
CYP51	2	2	1	0	1	0	10	2
**CYP72 clan**								
CYP72	12	18	7	1	9	1	13	4
**CYP709**	**0**	**0**	**1**	**0**	**3**	**0**	**9**	**3**
CYP714	6	12	3	0	2	0	5	1
CYP715	6	1	1	0	1	0	1	3
CYP721	2	5	1	0	1	0	2	0
CYP734	3	0	1	0	1	0	4	1
CYP735	3	3	1	0	1	0	2	0
CYP749	0	0	0	0	0	0	0	0
**CYP74 clan**								
CYP74	6	11	4	0	2	0	4	1
**CYP85 clan**								
CYP85	5	7	1	0	2	0	1	0
CYP87	2	2	2	0	1	1	11	2
CYP88	3	1	3	0	2	0	1	0
CYP90	12	5	4	0	4	0	5	1
**CYP702**	**0**	**0**	**0**	**0**	**6**	**3**	**0**	**0**
CYP707	10	6	3	0	4	0	3	0
**CYP708**	**0**	**0**	**0**	**0**	**4**	**0**	**0**	**0**
CYP716	7	4	3	0	2	0	0	0
CYP718	1	1	0	0	1	0	0	0
CYP720	2	3	1	0	1	0	0	0
CYP722	2	2	1	0	1	0	1	0
CYP724	1	2	0	0	1	0	1	0
CYP725	0	0	0	0	0	0	0	0
**CYP728**	**2**	**2**	**0**	**0**	**0**	**0**	**11**	**4**
CYP729	0	0	1	0	0	0	2	0
**CYP733**	**3**	**4**	**0**	**0**	**0**	**0**	**1**	**0**
**CYP86 clan**								
CYP86	9	26	3	0	11	0	5	6
CYP94	14	7	4	0	6	1	18	8
CYP96	7	7	5	1	13	2	12	0
CYP704	5	15	14	4	3	0	7	0
**CYP97 clan**								
CYP97	5	11	4	0	3	0	3	0
**CYP710 clan**								
CYP710	2	17	1	0	4	0	4	2
**CYP711 clan**								
CYP711	4	2	2	1	1	0	5	0
**CYP727 clan**								
**CYP727**	**1**	**0**	**0**	**0**	**0**	**0**	**1**	**0**
**CYP746 clan**								
CYP746	0	0	0	0	0	0	0	0
**Others**								
**Total**	**137**	**176**	**72**	**7**	**91**	**8**	**142**	**38**
