# Supplementary material for: Whole genome co-expression analysis of soybean cytochrome P450 genes identifies nodulation-specific P450 monooxygenases
Source: BMC Plant Biol. 2010 Nov 9;10:243. doi: 10.1186/1471-2229-10-243 (PMC3095325; doi:10.1186/1471-2229-10-243)
Supplement: Additional file 2 — Figure S1 Phylogenetic tree of all soybean P450s. S1A. A-type P450s of soybean and Arabidopsis. S1B. Non-A type P450s of soybean and Arabidopsis. Soybean P450s are shown in blue and Arabidopsis P450s are shown in green. Trees were constructed using MEGA4. [file 1471-2229-10-243-S2.PDF]

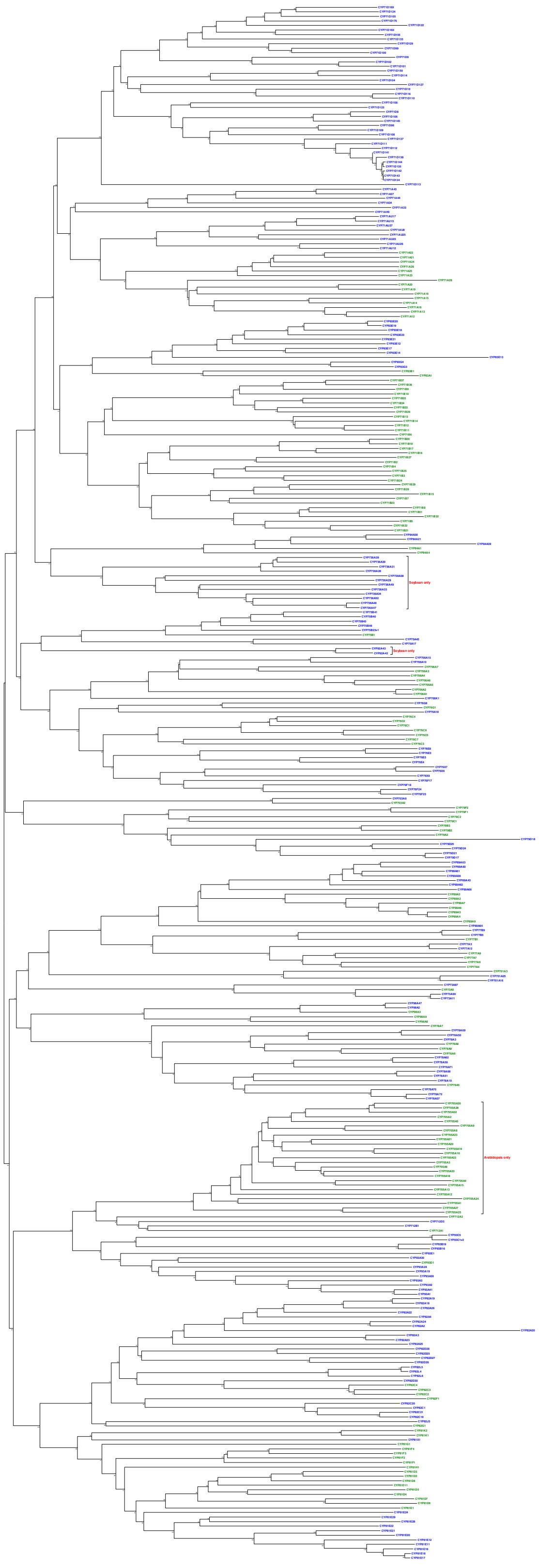

Fig1a. Neighbor-Join bootstrap tree of the A-type P450s. Soybean P450s are shown in blue and Aarabidopsis P450 are shown in green. Bootstrap values are out of 1000 replicates.

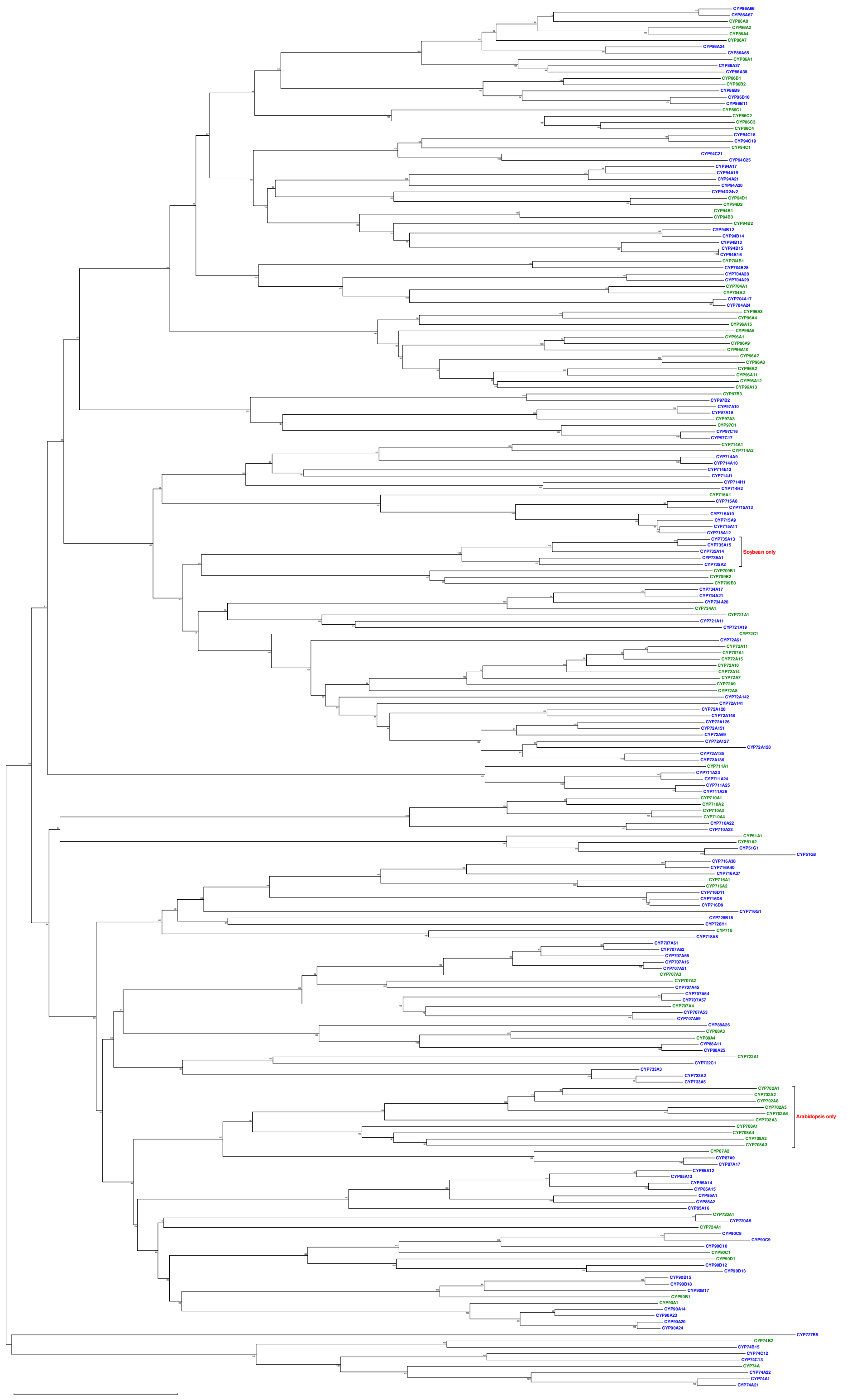

Fig1B. Neighbor-Join bootstrap tree of the Non-A type P450s. Soybean P450s are shown in blue and Aarabidopsis P450 are shown in green. Bootstrap values are out of 1000 replicates.
