# Supplementary material for: Whole genome co-expression analysis of soybean cytochrome P450 genes identifies nodulation-specific P450 monooxygenases
Source: BMC Plant Biol. 2010 Nov 9;10:243. doi: 10.1186/1471-2229-10-243 (PMC3095325; doi:10.1186/1471-2229-10-243)
Supplement: Additional file 5 — Figure S2 Gene expression of 332 soybean P450 genes according to the analysis of Illumina transcriptome dataset. Gene expression in different organ and root hair inoculation process. [file 1471-2229-10-243-S5.PDF]

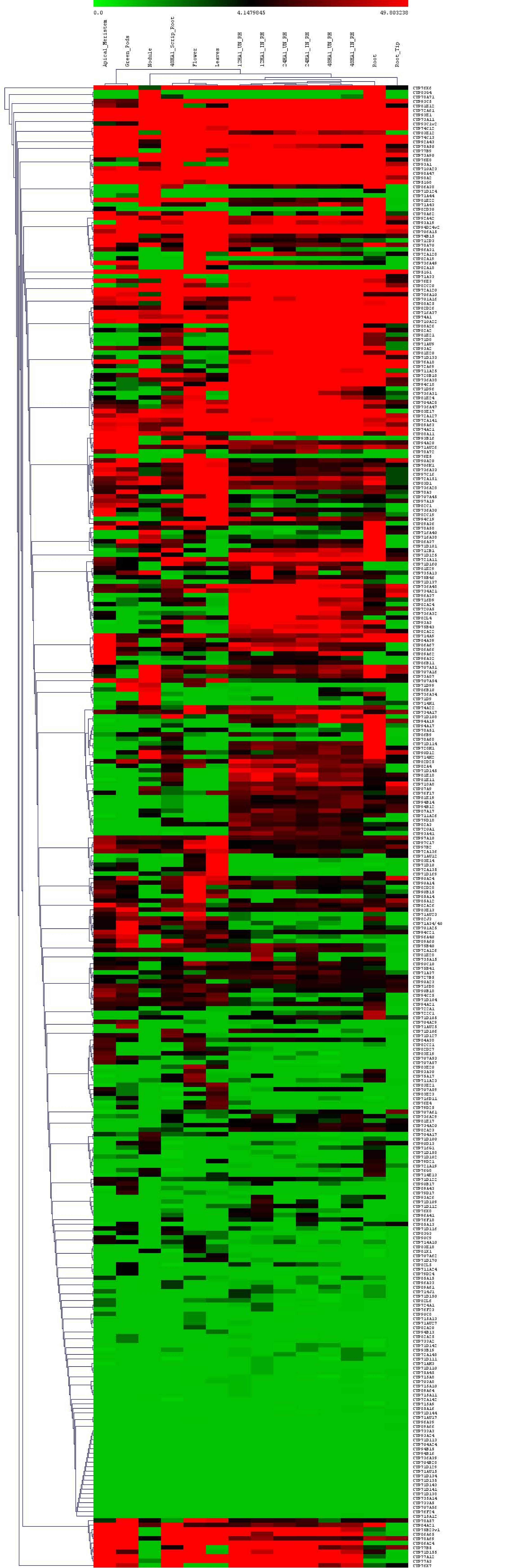
0.0
4.1479845
49.803238
Apical_Meristem
Green_Pods
Nodule
48HAI_Scrip_Root
Flower
Leaves
12HAI_UN_RH
12HAI_IN_RH
24HAI_UN_RH
24HAI_IN_RH
48HAI_UN_RH
48HAI_IN_RH
Root
Root_Tip
