# Supplementary material for: Whole genome co-expression analysis of soybean cytochrome P450 genes identifies nodulation-specific P450 monooxygenases
Source: BMC Plant Biol. 2010 Nov 9;10:243. doi: 10.1186/1471-2229-10-243 (PMC3095325; doi:10.1186/1471-2229-10-243)
Supplement: Additional file 7 — Table S5 List of primer pairs used in qRT-PCR [file 1471-2229-10-243-S7.PDF]

Table.S5. List of Primer pairs used in quantitative RT-PCR

Gene name	Forward Primer	Reverse primer
CYP78A71	5'-TGGTTGGTGAAATGGTGATG-3'	5'-CCTCTACCACCCCTTTGTCC-3'
CYP83G4	5'-CAGCTTCCACCTTCTACACAGA-3'	5'-CGACCCATCAACCCAGTG-3'
CYP71A44	5'-AGGCATTGACATTGTTTTTGG-3'	5'-CAGAAGTTCACGGGCACATA-3'
CYP82D30	5'-ACATCTGGCTCGGCACTC-3'	5'-TGGATGTGAAACATTCTTTTGC-3'
CYP81E12	5'-AACCTCTCTCGACGTTCTATCG-3'	5'-GCCTGTGTATCAACCTTTTCG-3'
CYP736A34	5'-AATATCAAGGCTATTTTGTTGGATATG-3'	5'-CCACTCAACCCCAATAGCAG-3'
CYP81E28	5'-AAACCCATGTGGGACAAGAC-3'	5'-GGTAAGGGAGTTTTGAAAGGTCA-3'
CYP83E14	5'-TCCAACTAATTCCTTTTGGAACA-3'	5'-TTCTAATATCACAACTGCCATAGGTAA-3'
CYP82D26	5'-CGGTGCTGCAACAAGAAAA-3'	5'-GGTGGATGTGACCAATTAAAGG-3'
CYP74B15	5'-GAAGCCATCCACAATTTGCT-3'	5'-AATGGAGAATCCGCCGTAG-3'
CYP93C5	5'-GCTCATCAACAATCCCAG-3'	5'-ACTTCGTCAACGAGTCTA-3'
CYP83E21	5'-TTCAAAGAGACCCTGAAATATGG-3'	5'-TGTATAAGAACCTCTCAGGCAAAA-3'
